# Selective Generation of Lamb Wave Modes in a Finite-Width Plate by Angle-Beam Excitation Method

**DOI:** 10.3390/s20143868

**Published:** 2020-07-10

**Authors:** Sang-Jin Park, Young-Sang Joo, Hoe-Woong Kim, Sung-Kyun Kim

**Affiliations:** 1 Versatile System Technology Development Division, Korea Atomic Energy Research Institute, 111 Daedeok-daero 989beon-gil, Yuseong-gu, Daejeon 34057, Korea; sjpark@kaeri.re.kr (S.-J.P.); hwkim@kaeri.re.kr (H.-W.K.); sungkyun@kaeri.re.kr (S.-K.K.); 2 Department of Advanced Nuclear System Engineering, Korea University of Science and Technology, 217 Gajeong-ro, Yuseong-gu, Daejeon 34113, Korea

**Keywords:** Lamb wave, finite-width plate, higher-order width mode, angle-beam excitation method, SAFE (semi-analytical finite element) technique, mode selection

## Abstract

A Lamb wave in a plate with a finite width has both thickness and width modes, whereas only thickness modes exist in an infinitely wide plate. The thickness and width modes are numerously formed in a finite-width plate, and they all have different cut-off frequencies, wave velocities, and wave structures. These different characteristics can be utilized in various applications, but a selective generation method for a particular Lamb wave mode in a finite-width plate has not been sufficiently studied, and only a method using multiple elements has been reported. This paper presents the selective generation of a certain Lamb wave mode in a finite-width plate by an angle-beam excitation method using single or dual wedges. In the proposed generation method, a specially designed wedge with grooves or a patch having insulation layers is employed for partial acoustic insulation of the ultrasonic energy incident into the plate. The feasibility of the proposed method was investigated through finite element method (FEM) simulations for Lamb wave excitation and propagation, and then experimentally demonstrated by the measurement of Lamb wave propagation using a laser scanning vibrometer.

## 1. Introduction

Lamb waves are elastic guided waves that have thickness modes formed by top and bottom boundaries of an infinitely wide plate. They can propagate a long distance along the plate, and their lowest-order modes are particularly sensitive to defects at any location in the thickness direction, including surface cracks. These characteristics have been effectively utilized in nondestructive testing (NDT) and structural health monitoring (SHM) of huge plate-like structures [1,2].

In NDT and SHM based on Lamb wave technology, it is important to properly design the operational frequency and the wave mode. In particular, the wave mode should be appropriately selected to have optimum performance suitable for the application purpose. For instance, when examining fluid-coupled structures, some applications choose the extensional wave modes to avoid the degradation of the long-distance propagation ability by energy leakage [3,4]. But conversely, the flexural wave modes are preferred to utilize the high energy leakage [5] in other applications. In addition, Lamb wave modes have been selected to achieve the best detectability and resolution for various types of imperfections such as surface cracks [6], wall thinning [7], and icing detection [8]. Therefore, mode selection is fundamental and essential in Lamb wave-based NDT and SHM. 

Fortunately, Lamb wave mode selection in a low-frequency range is easy, because there are only two lowest-order modes ($S_{0}$- and $A_{0}$-modes) that are far away from other higher-order modes. For this reason, these two Lamb wave modes have mainly been chosen for Lamb wave-based NDT and SHM. However, such facile mode selection of the fundamental modes is no longer valid in a finite-width plate, because modal density increases by forming additional wave modes confined by both lateral boundaries of the plate. Hence, the Lamb wave in a finite-width plate needs to be treated more sensitively. A finite-width plate is often used at the waveguide sensor for inspection and sensing in hazardous environments [9,10,11,12], such as in the case of a plate-type ultrasonic waveguide sensor for under-sodium viewing in a sodium-cooled fast reactor. Without any damage to the main actuating unit, this waveguide sensor can remotely perform ultrasonic visualization under hot liquid sodium (>200 °C) by using the leaky Lamb wave theory [11,12].

Long strip-like structures such as rails, H-beams, and rectangular beams have also been widely used in many industrial applications. In these finite-width plate structures, thickness and width modes of the Lamb wave are numerously formed, and they all have different cut-off frequencies, wave velocities, and wave structures. These different characteristics enable varied utilization in many applications. However, in most applications, Lamb wave propagation in a finite-width plate has been conventionally employed without consideration of the effects of the width modes. Moreover, the selective generation method for a certain Lamb wave mode in a finite-width plate has not been sufficiently investigated.

A few studies have been conducted to selectively generate the Lamb wave mode in a finite-width plate. In one study, a selective excitation method using multiple point sources was numerically studied, and it was found that the performance of the proposed method varied with the number of point sources [13]. In another study, a mode selection method using phased array was investigated [14]. The reported phased-array method selectively generated the target Lamb wave mode by using eight piezo-elements. However, the phased-array method required multiple elements and multi-channel devices as well as a control technique of the input signal for each element.

In this paper, an angle-beam excitation method using single or dual wedges is proposed for the selective generation of a certain Lamb wave mode in a finite-width plate. Unlike the phased-array method, the proposed method does not require multi-channel devices or control of the input signal. In addition, the angle-beam excitation method generates Lamb waves in a single direction, which might be advantageously used in NDT and SHM. The feasibility of the proposed method was investigated through finite element method (FEM) simulations of Lamb wave excitation and propagation, and then experimentally demonstrated by the measurement of Lamb wave propagation using a laser scanning vibrometer.

## 2. Lamb Wave Propagation Characteristics in A Finite-Width Plate

### 2.1. The SAFE Technique

**Figure 1:**
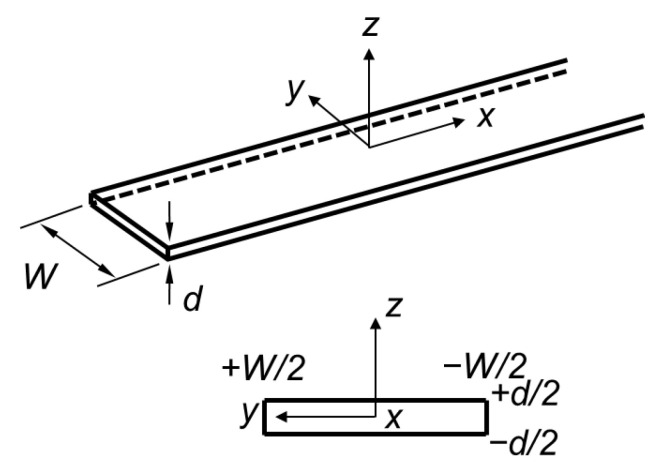
Coordinate system of a finite-width plate.

Before discussing the selective generation method of the Lamb wave mode, it is important to understand the propagation behavior of a Lamb wave in a finite-width plate, as illustrated in Figure 1. The finite-width plate has width W and thickness d, and its length is infinite in the ±x direction. Lamb wave propagation in a finite-width plate has been analytically studied by multiple researchers [15,16,17], and a numerical method, called SAFE (Semi-Analytical Finite Element), has been widely employed to calculate the dispersion curves of a Lamb wave in a complex structure with an arbitrary cross section including a finite-width plate [18,19,20]. 

The SAFE technique models the cross section of the analysis object as the finite elements, and then calculates the solution of the constructed system by adopting the analytical solution of the wave propagation. The governing equation of the constructed finite element system can be expressed as:

(1) $$\left\{\left[K_{1}\right]+i\gamma \left[K_{2}\right]+\gamma^{2}\left[K_{3}\right]+\omega^{2}\left[M\right]\right\}\left\{u\right\}=0$$

where $\left[K_{1}\right]$, $\left[K_{2}\right]$, and $\left[K_{3}\right]$ are stiffness matrices, [M] is a mass matrix of the cross section, and {u} is a displacement vector. The displacement vector {u} can be given by the analytical solution of the harmonic function,

(2) $$\left\{u\right\}=\{U\left(y,z\right)\}e^{i\left(\gamma x-\omega t\right)}$$

where {U(y,z)} is a deformation shape vector. From this eigenvalue equation, the wavenumber γ can be solved at a given frequency ω, and the deformation shape can also be obtained. The dispersion curve can be drawn from solving iterations within the interested frequency range. The SAFE technique using commercial FEM codes has been reported [21,22]. 

### 2.2. Dispersion Curves And Wave Structures

**Figure 2:**
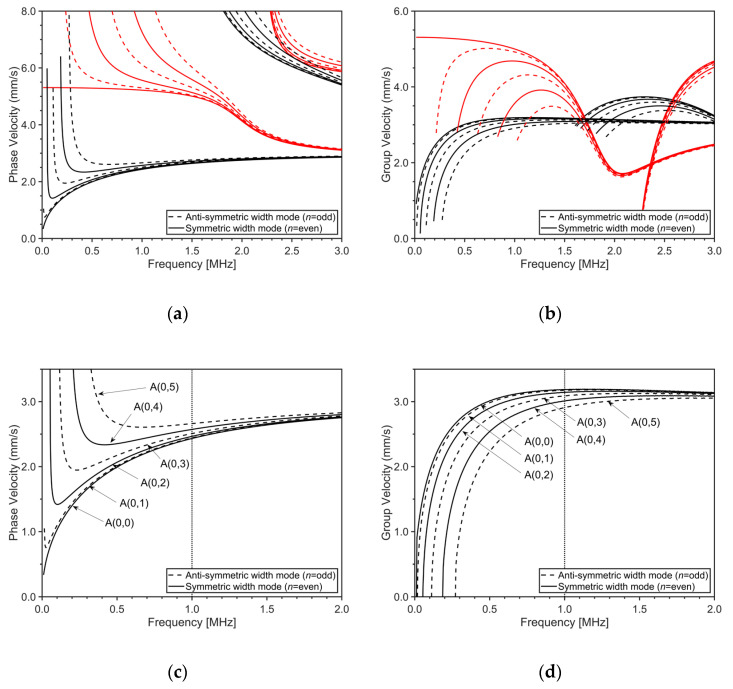
Dispersion curves of Lamb waves in SS304 plate (ρ = 7800 kg/m^3^, $C_{L}$ = 5800 m/s and $C_{S}$ = 3160 m/s) with thickness of 1.2 mm and width of 15 mm: (**a**) phase velocity dispersion curves, (**b**) group velocity dispersion curves, (**c**) phase velocity dispersion curves of A(0,*n*) modes, and (**d**) group velocity dispersion curves of A(0,*n*) modes; black line: anti-symmetric thickness mode, red line: symmetric thickness mode.

Figure 2 shows the dispersion curves of Lamb waves in an SS304 plate with 1.2 mm thickness and 15 mm width calculated by the SAFE technique based on axisymmetric modal analysis [22] using the commercially available FEM code, ANSYS (release 2017, ANSYS Inc., Canonsburg, PA, USA). From the dispersion curves, it can be seen that additional wave modes are formed with the existing Lamb wave modes in the infinite plate. For example, $S_{0}$- and $A_{0}$-modes, the fundamental Lamb wave modes in the infinite plate, are no longer alone in the finite-width plate. Accordingly, a certain Lamb wave mode in a finite-width plate is called S(*m*,*n*) or A(*m*,*n*); the first index *m* is the order of the thickness mode, and the second index *n* is the order of the width mode. Also, it can be seen in the dispersion curves that the phase velocities of higher-order width modes increase, while the group velocities decrease in the entire frequency range as the order of the width mode increases. Figure 2c,d are the dispersion curves of A(0,*n*) modes, separately plotted from Figure 2a,b, respectively. From the figures, it can be seen that A(0,*n*) modes are very close to each other; although they are relatively far away from each other in the low frequency range, the modal density increases as the frequency increases. From this trend, it can be supposed that it is difficult to selectively excite a single particular Lamb wave mode in a finite-width plate.

**Figure 3:**
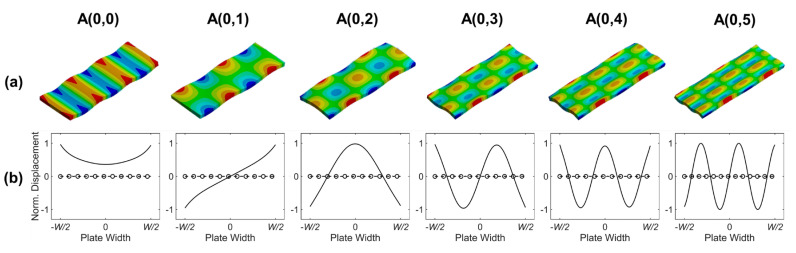
Wave structures of A(0,*n*) modes in SS304 plate with 1.2 mm thickness and 15 mm width at 1.0 MHz: (**a**) wave structures of A(0,*n*) modes and (**b**) displacement distributions in the width direction on the neutral plane(*z* = 0); solid line: out-of-plane direction (*z* direction), dashed line: in-plane horizontal direction (*y* direction), and circle markers: in-plane extensional direction (*x* direction).

Figure 3 depicts the wave structures of the A(0,*n*) modes. The odd order modes show the anti-symmetric out-of-plane displacement distributions in the width direction, whereas the even order modes show the symmetric out-of-plane displacement distributions. 

## 3. Angle-Beam Excitation Method for Selective Generation of Lamb Wave Mode

### 3.1. Selective Excitation Method Concept

For the selective excitation of a certain Lamb wave mode in a finite-width plate, unlike the infinitely wide plate case where only the wavelength of the target mode in the propagation direction is considered, the wavelength of the target mode in the width direction should also be considered. In other words, for a certain width mode selection, the profile of the ultrasonic energy incident into the plate should correspond to the wave structure of the target mode in the width direction. To this end, a specially designed wedge is employed in the proposed angle-beam excitation method. Based on the displacement distributions shown in Figure 3b, the proposed method acoustically insulates the negative or positive displacement regions of the wave structure.

**Figure 4:**
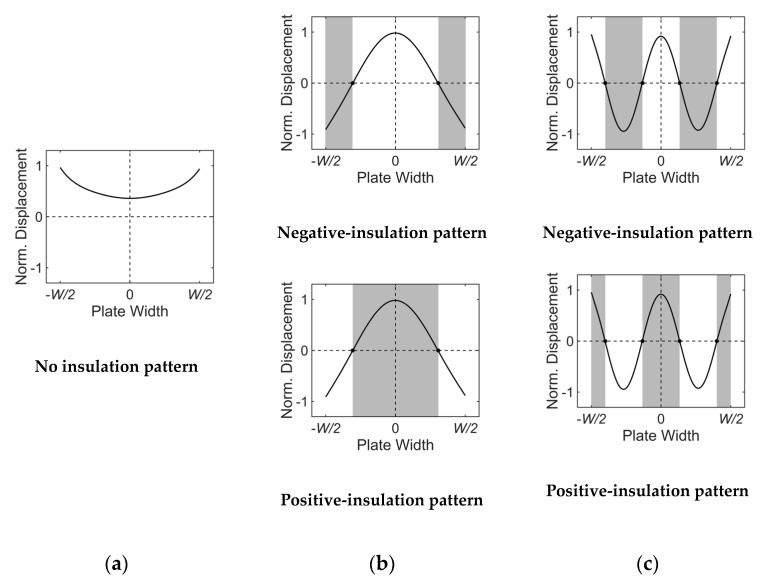
Acoustically insulated regions for symmetric width modes: (**a**) A(0,0), (**b**) A(0,2), (**c**) A(0,4) modes; the shadowed are acoustically insulated regions.

**Figure 5:**
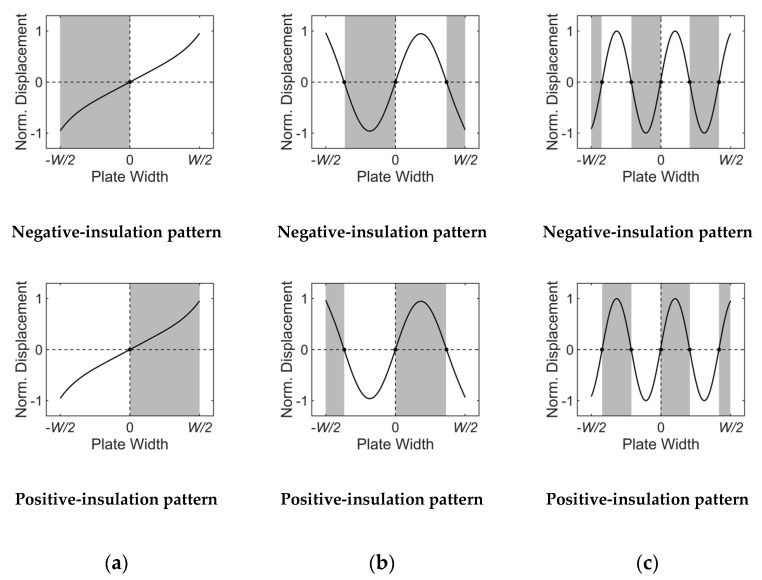
Acoustically insulated regions for anti-symmetric width modes: (**a**) A(0,1), (**b**) A(0,3), (**c**) A(0,5) modes; the shadowed are acoustically insulated regions.

Figure 4 and Figure 5 present acoustically insulated regions based on the proposed method for the selective generation of A(0,0)–A(0,5) modes. The generation of the A(0,0) mode does not require acoustic insulation because its displacement is in-phase in the width direction, as shown in Figure 4a. Therefore, the A(0,0) mode can be sufficiently generated by using a conventional wedge. Unlike the A(0,0) mode, other higher-order width modes require acoustic insulation for selective generation. Figure 4b,c show acoustically insulated regions for the selective generation of A(0,2) and A(0,4) modes, respectively. In these symmetric width mode cases, two different acoustic insulation patterns can be adopted; one for the insulation of the negative displacement region, called the negative-insulation pattern, and the other for the insulation of the positive displacement region, called the positive-insulation pattern. Acoustically insulated regions for the selective generation of A(0,1), A(0,3), and A(0,5), which are anti-symmetric width modes, are shown in Figure 5a–c, respectively. For the anti-symmetric width mode, two acoustic insulation patterns can also be adopted but they are symmetric to the central axis in the width direction. 

### 3.2. Wedge Designs

**Figure 6:**
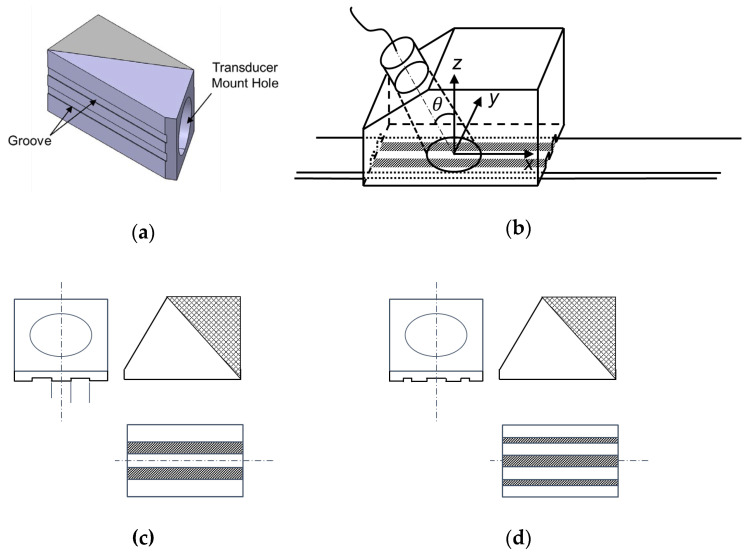
(**a**) Groove-based wedge and (**b**) its working principle; the shadowed are insulated regions. Groove-based wedges for A(0,4) mode with (**c**) negative-insulation pattern and (**d**) positive-insulation pattern.

Based on the acoustic insulation technique presented in Section 3.1, two types of wedge designs, groove- and patch-based wedges, are introduced in this paper. The groove-based wedge has engraved grooves on its bottom face, as shown in Figure 6. Since the ultrasonic energy generated from the ultrasonic transducer cannot be incident into the plate at grooves, the profile of the excitation input can be corresponded with the wave structure of the target wave mode in the width direction. Note that a shallow groove is recommended to avoid wave interference caused by reflection and mode conversion from the groove. 

**Figure 7:**
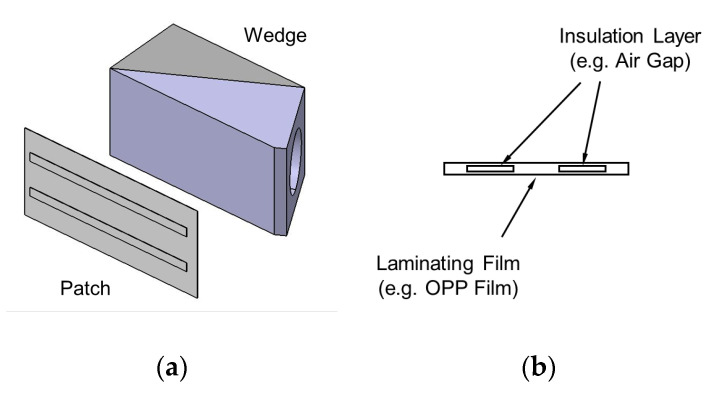
(**a**) Patch-based wedge and (**b**) cross section of the patch.

The patch-based wedge employs a patch having acoustic insulation layers in its inside, as illustrated in Figure 7. The material for the acoustic insulation layer should have a big difference in acoustic impedance from the plate and the wedge, such as with an air gap. A thin film (e.g., OPP [Oriented PolyPropylene] film) is recommended to avoid acoustic attenuation in the patch. The patch should be placed between the wedge and the plate to match the profile of the excitation input with the wave structure of the target wave mode in the width direction; it can be attached to the bottom face of the wedge or the top surface of the plate. The patch-based wedge does not require any modification of the common wedge and allows the best excitation conditions for the given target wave mode by switching the patch having proper acoustic insulation layers. 

### 3.3. Single and Dual Wedge Techniques

**Figure 8:**
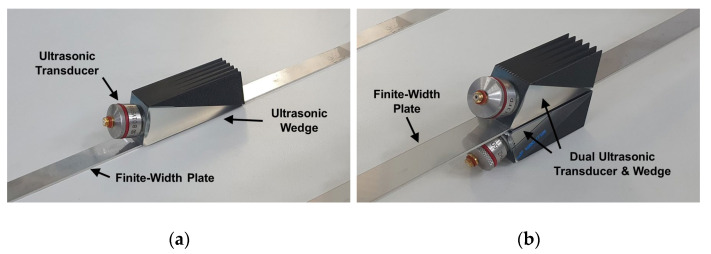
(**a**) Single wedge technique and (**b**) dual wedge technique.

For the generation of a certain Lamb wave mode, a single or a dual wedge technique can be used in the proposed method. The single wedge technique uses one set of a transducer and a wedge mounted on the plate, as shown in Figure 8a; this technique is exactly the same as the conventional angle-beam excitation method. In this single wedge technique, either of the two types of insulation patterns can be used.

Two types of insulation patterns can be individually used in the single wedge technique, but they can be used simultaneously in the dual wedge technique. The dual wedge technique uses two sets of a transducer and a wedge mounted on the top and bottom surfaces of the plate, as shown in Figure 8b. Since the ultrasonic energy can be alternately incident into the top and bottom surfaces of the plate in the width direction through two different types of insulation patterns, the profile of the excitation input can be more similar to the wave structure of the target wave mode in the dual wedge technique. For the dual wedge technique, a single- or multi-channel pulser can be used because it is not necessary to control the input signal at each channel in the proposed selective excitation method. By branching the input signal from a single-channel pulser, the same input signals can be sent into two transducers. 

## 4. FEM Simulation for Selective Generation of Lamb Wave Mode

**Figure 9:**
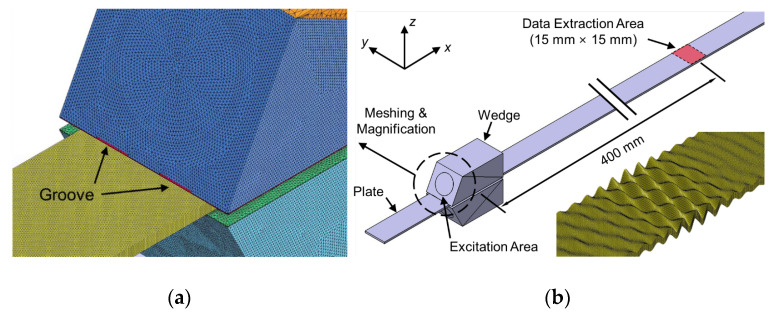
(**a**) Meshed FEM model and (**b**) overview of the entire FEM model and propagation snapshot of the excited A(0,4) mode.

A FEM simulation model consisting of a finite-width plate and two groove-based wedges was constructed to investigate the feasibility of the proposed selective generation method, as shown in Figure 9. It was a transient analysis model, and the simulation was performed using the commercially available FEM software, ANSYS/LS-DYNA (release 2017, ANSYS Inc., Canonsburg, PA, USA). SS304, the same material used in the SAFE model, was used for the plate, whose thickness, width, and length were 1.2 mm, 15 mm, and 1000 mm, respectively. The material for the wedge was Lucite ($C_{L}$ = 2370 m/s), and the width and height of the wedge were 20 mm and 19 mm, respectively. The depth of the groove at the bottom of the wedge was 0.3 mm. The incidence angle of the wedge was changed depending on the target wave mode, and the diameter of the excitation area was 1/2 inch (12. 7 mm). A four-cycle tone burst centered on 1.0 MHz was used as the input signal and applied to the excitation area as the displacement. Note that a tone burst is better than a spike pulse for mode selection in a high modal density range because a spike pulse has a wide frequency band. The selective excitation and propagation simulations for modes A(0,0)–A(0,5) were performed based on the dual wedge technique with groove-based wedges; the total simulation time was 165 μsec and the integration time step was 50 nsec. Here, simulations for the A(0,0) mode were conducted based on the single wedge technique using a common wedge without grooves, because the generation of the A(0,0) mode did not require acoustic insulation, as shown in Figure 4a. The finite-width plate was meshed by 0.15 mm-sized hexahedron elements, and the wedge was meshed by 0.3 mm-sized tetrahedron elements, as shown in Figure 9a. To investigate the feasibility of the proposed method, out-of-plane displacements around 400 mm away from the excitation source were extracted, as represented in Figure 9b; the extraction area was 15 mm × 15 mm and the extraction interval was 0.3 mm. 

**Figure 10:**
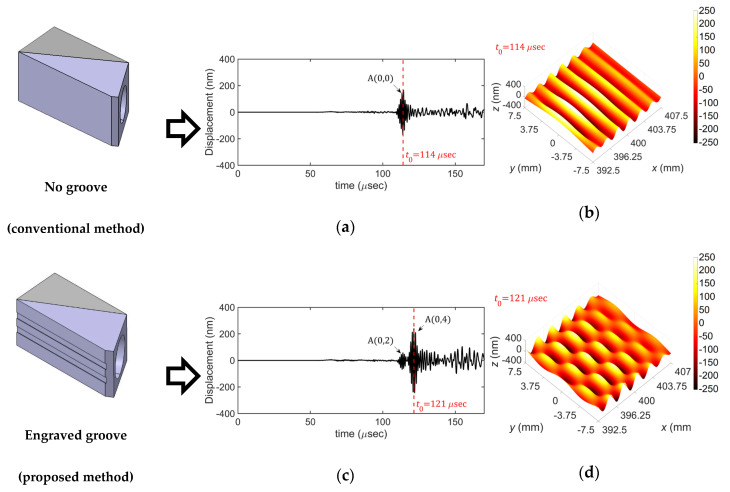
FEM simulation results of the conventional and the proposed wedges for excitation of the A(0,4) mode, (**a**) time history and (**b**) displacement distribution obtained by using the conventional wedge, (**c**) time history and (**d**) displacement distribution obtained by using the proposed wedge; time histories were picked up at side edge at 400 mm away from the excitation source.

Figure 10 compares the FEM simulation results for the selective excitation of the A(0,4) mode based on the conventional method and the proposed method. Figure 10a,b show the time history of the out-of-plane displacement at the side edge at 400 mm away from the excitation source and the displacement distribution extracted from the extraction area at *t* = *t*_0_ obtained by using the common wedge without grooves while those obtained by using the groove-based wedge are shown in Figure 10c,d. From the results, it can be clearly seen that the target wave mode, A(0,4) mode, cannot be generated by the conventional method whereas it is well generated by the proposed method. 

**Figure 11:**
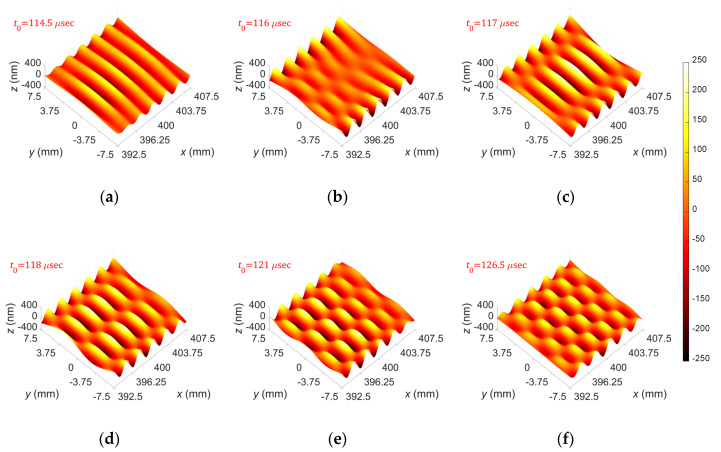
FEM simulation results: displacement distributions of (**a**) A(0,0), (**b**) A(0,1), (**c**) A(0,2), (**d**) A(0,3), (**e**) A(0,4), and (**f**) A(0,5) modes.

Figure 11 shows displacement distributions of the A(0,0)–A(0,5) modes obtained from the performed FEM simulations based on the proposed excitation method; the displacement distributions were extracted from the extraction area at *t* = *t*_0_. From these results, one can see that the proposed selective excitation method well generates the target wave mode in the finite-width plate.

## 5. Experiments

### 5.1. Experimental Setup

**Figure 12:**
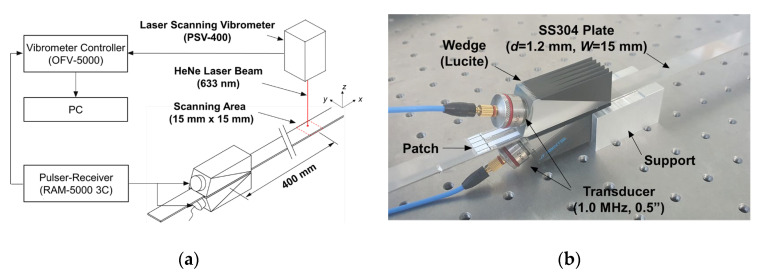
(**a**) Schematic diagram of the experimental setup and (**b**) installed patch-based wedges for Lamb wave propagation measurement in a finite-width plate using a laser scanning vibrometer.

Figure 12 shows a schematic diagram of the experimental setup and installed patch-based wedges for Lamb wave propagation measurement in a finite-width plate using a laser scanning vibrometer (POLYTEC PSV-400). As shown in Figure 12b, two patch-based wedges were installed at the top and bottom surfaces of the plate for the dual wedge technique. Again, the single wedge technique using a common wedge mounted on the top surface of the plate was used for the A(0,0) mode excitation, because the generation of the A(0,0) mode did not require acoustic insulation, as mentioned previously. The ultrasonic transducer used in the experiment was a commercial piezoelectric transducer (GE Benchmark series) with a diameter of 1/2 inch (12.7 mm) and a center frequency of 1.0 MHz. The ultrasonic wedge material was Lucite ($C_{L}$= 2370 m/s), and the wedge dimensions were the same as those in the FEM simulation model. A 0.3 mm-thick OPP laminating film patch having acoustic insulation layers (0.1 mm-thick paper) in its inside was installed between each wedge and the plate. Note that the incidence angle of the wedge and the patch were properly selected depending on the target wave mode. Also, the material and the dimensions of the finite-width plate used in the experiment were the same as those in the FEM simulation model. As an input signal, a four-cycle tone burst generated from a 3-channel high power pulser/receiver (RITEC RAM-5000 3C) was sent to each transducer with the same amplitude and phase. As in the FEM simulation, the out-of-plane (*z*-axis) displacements of A(0,0)–A(0,5) modes around 400 mm away from the excitation source were measured by the laser scanning vibrometer; the scanning area was 15 mm × 15 mm and the scanning interval was 0.3 mm. The sampling frequency of the vibrometer was 25.6 MHz and the total sampling number was 8192. Then, measured signals were filtered through a high-pass filter (0.5 MHz cut-off frequency). For comparison between the groove-based and the patch-based wedges in selective excitation performance, additional experiments using the groove-based wedges were also carried out for the selective excitation of the A(0,4) mode. 

### 5.2. Measurement Results

**Figure 13:**
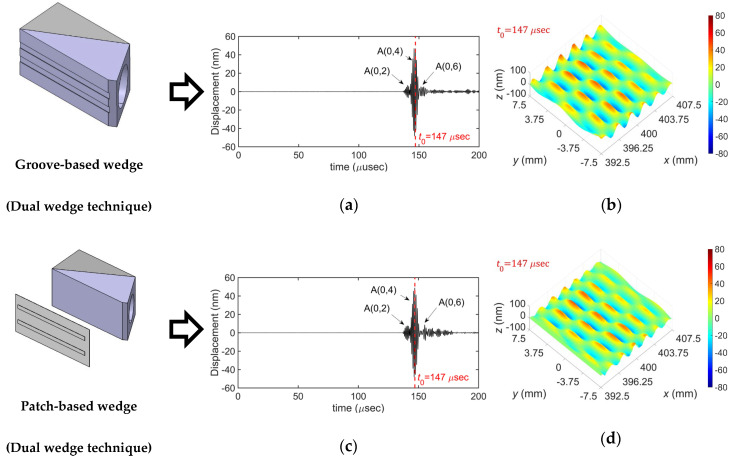
Experimental results for the selective generation of the A(0,4) mode: (**a**) time history and (**b**) displacement distribution obtained by using the groove-based wedge; (**c**) time history and (**d**) displacement distribution obtained by using the patch-based wedge; time histories were picked up at side edge at 400 mm away from the excitation source.

Figure 13 compares the experimental results for the selective generation of the A(0,4) mode obtained by using the dual wedge technique with the groove-based and patch-based wedges. The amplitude of each measured signal at *t* = *t*_0_ was extracted to obtain the displacement distribution. From the results, it can be seen that the target wave mode, A(0,4) mode, can be well generated by both types of wedges. It can also be seen that the mode selection performance of the groove-based wedge is slightly better than that of the patch-based wedge; the peak-to-peak amplitude of the A(0,4) mode is 17.5 dB greater than that of the A(0,2) mode in the groove-based wedge case, whereas it is 15.5 dB greater than that of the A(0,2) mode in the patch-based wedge case. This is thought to be caused by the fact that the groove-based wedge is directly coupled with the plate, whereas the patch-based wedge has an intermediate medium between the wedge and the plate. However, although the mode selection performance of the groove-based wedge is slightly better than that of the patch-based wedge, the patch-based wedge provides better usability because it can be used with a common wedge without any modification by switching the proper patch for the given target wave mode.

**Figure 14:**
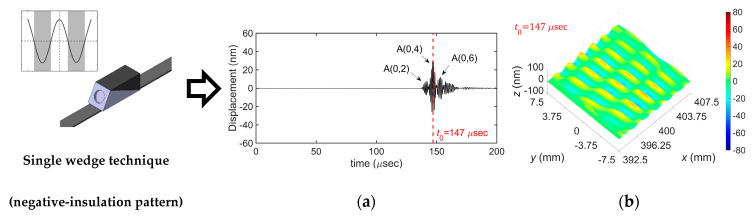

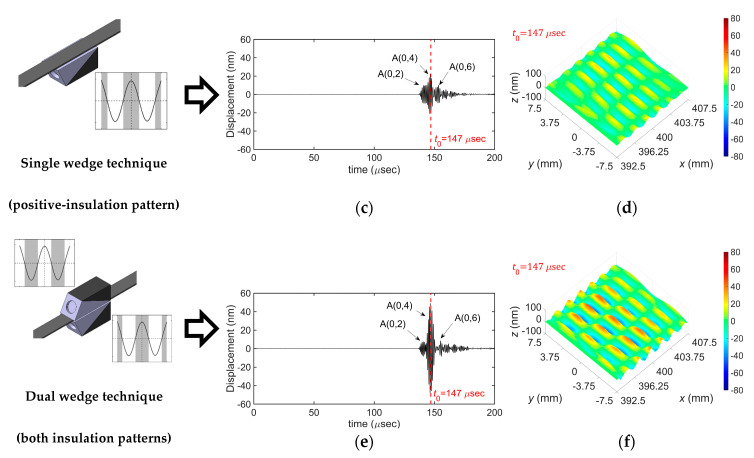
Experimental results for the selective generation of the A(0,4) mode: (**a**) time history and (**b**) displacement distribution obtained by using the single wedge technique with the negative-insulation pattern; (**c**) time history and (**d**) displacement distribution obtained by using the single wedge technique with the positive-insulation pattern; (**e**) time history and (**f**) displacement distribution obtained by using the dual wedge technique with both insulation patterns; time histories were picked up at side edge at 400 mm away from the excitation source.

Figure 14 compares the experimental results for the selective generation of the A(0,4) mode obtained by using single and dual wedge techniques with the patch-based wedge. Two types of insulation patterns, negative- and positive-insulation patterns, were used individually in the single wedge technique, whereas they were used simultaneously in the dual wedge technique. From the results, first, the mode selection performance of the dual wedge technique is much better than that of the single wedge technique. The peak-to-peak amplitude of the A(0,4) mode compared with that of the A(0,2) mode in the dual wedge case is 4 dB greater than that in the single wedge case; 15.5 dB in the dual wedge case and 11.5 dB in the single wedge case. Second, in the single wedge cases, the mode selection performance of the negative-insulation pattern is better than that of the positive-insulation pattern; the peak-to-peak amplitude of the A(0,4) mode is 11.5 dB greater than that of the A(0,2) mode in the negative-insulation pattern case, whereas it is 7.4 dB greater than that of the A(0,2) mode in the positive-insulation pattern case. This is thought to be caused by the radiation characteristics of the ultrasonic transducer, which has the maximum intensity at the central axis in the radiation direction. Since the negative-insulation pattern has the excitation area in the middle of the plate width, more intense energy can be incident into the plate compared with the positive-insulation pattern, which has the insulated region in the middle of the plate width. Hence, the negative-insulation pattern is recommended when the single wedge technique is used for the selective generation of a certain Lamb wave mode in the finite-width plate.

**Figure 15:**
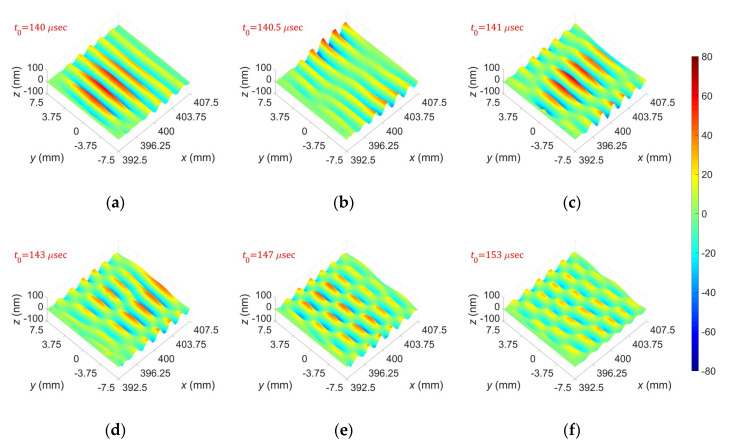
Experimentally obtained out-of-plane displacement distributions for (**a**) A(0,0), (**b**) A(0,1), (**c**) A(0,2), (**d**) A(0,3), (**e**) A(0,4), and (**f**) A(0,5) modes.

Figure 15 presents displacement distributions of A(0,0)–A(0,5) modes generated by the dual wedge technique using patch-based wedges. Similar to the results in Figure 13; Figure 14, the amplitude of each measured signal at *t* = *t*_0_ was extracted to obtain the displacement distribution. From the figure, one can see that the experimentally obtained displacement distributions agree well with the FEM simulation results in Figure 11, which means that the proposed selective excitation method well generates the target wave mode in a finite-width plate. 

As described above, the proposed method can selectively generate the target wave mode in the finite-width plate well with the simple positive- or negative-acoustic insulation pattern. But the proposed insulation pattern may not be the best one and one can consider its shape optimization to maximize the selective excitation efficiency. The optimization should be performed to match the input energy with the wave structure of the target wave mode more closely; non-flat design or dimension change of the insulation layer can be one of optimization examples.

## 6. Conclusions

This paper proposed a selective generation method of a certain Lamb wave mode in a finite-width plate using an angle-beam excitation technique, which is easy and simple compared with, for example, a phased-array technique. The proposed method employs acoustic insulation for matching the profile of the ultrasonic energy incident into the plate with the wave structure of the target wave mode. For the acoustic insulation, two types of wedge designs were introduced: a groove-based wedge that has engraved grooves on its bottom face and a patch-based wedge that has a thin patch with acoustical insulation layers in its inside. The feasibility of the proposed selective generation method was investigated through FEM transient analyses for Lamb wave excitation and propagation, and then it was experimentally demonstrated by Lamb wave propagation measurements using a laser scanning vibrometer. From the simulation and experimental results, it was validated that the proposed method could selectively generate the target Lamb wave mode in a finite-width plate. Furthermore, it was observed that the dual wedge technique provided better results than the single wedge technique in mode selection performance, and a negative-insulation pattern is recommended in the case of the single wedge technique. As future work, further studies on the combination, filtering, and controlling of a Lamb wave in a finite-width plate will be undertaken based on the proposed method.
